# Analysis of the effect of BMI on depression and anxiety among older adults in China: the mediating role of ADL and IADL

**DOI:** 10.3389/fpubh.2024.1387550

**Published:** 2024-09-04

**Authors:** Ziqing Qiao, Zichun Wang, Jiaping Qiu, Jie Zhang, Weiyi Cao

**Affiliations:** ^1^Nanqiao Town Community Health Service Center, Shanghai, China; ^2^Shanghai Fengxian District Center for Disease Control and Prevention, Shanghai, China

**Keywords:** BMI, depressive symptoms, anxiety symptoms, mediation effect, ADL, IADL

## Abstract

**Background:**

Anxiety and depression are serious psychological and public health issues among the older adults. Currently, there is a lack of understanding of the relationship between Body Mass Index (BMI) and anxiety or depression symptoms in the older adult population in China. Therefore, the purpose of this study is to investigate the impact of BMI on anxiety and depression through correlation analysis in different subgroups and to examine the potential chain mediating effects of Activities of Daily Living (ADL) and Instrumental Activities of Daily Living (IADL) between BMI and symptoms of anxiety and depression.

**Methods:**

From the CLHLS database conducted in 2017–2018, data regarding height, weight, anxiety symptoms, depression symptoms, as well as demographic, socioeconomic, behavioral, and health-related characteristics were collected. Multivariate logistic regression analysis was used to explore the impact of BMI on anxiety and depression symptoms. Finally, the SPSS macro process was utilized to test the multiple mediating effects of ADL and IADL between BMI and anxiety or depression symptoms.

**Results:**

After screening, a final sample of 9,098 Chinese older adult individuals aged 65 and above was selected. Among them, 1,074 cases (11.8%) exhibited anxiety symptoms, 1,458 cases (16.0%) exhibited depressive symptoms. Compared to normal BMI, the adjusted analysis showed that underweight in Chinese older adult individuals was significantly associated with anxiety (OR = 1.316, *p* = 0.004) and depression (OR = 1.232, *p* = 0.013). This relationship was found to be more significant in males, individuals aged 80 and above, unmarried individuals, and those residing in town. ADL and IADL played a chain-mediated role between BMI and anxiety symptoms in the older adult. BMI not only had a direct effect on anxiety symptoms in the older adult (effect = −0.0159; SE = 0.0066; 95%CI: LL = −0.0288, UL = −0.0031), but also influenced them indirectly through two pathways: the independent mediating role of IADL (effect = −0.0010; SE = 0.0005; 95%CI: LL = −0.0018, UL = −0.0003) and the chain-mediated role of ADL and IADL (effect = −0.0012; SE = 0.0004; 95%CI: LL = −0.0020, UL = −0.0006).

**Conclusion:**

In the older adult individuals in China, underweight is associated with an increased risk of anxiety and depression symptoms. Improving the underweight condition of Chinese older adult individuals can reduce their ADL and IADL limitations, thereby benefiting their mental health.

## 1 Introduction

Anxiety and depression are prevalent among older adults, posing significant psychological and public health challenges for this age group and being leading causes of disability in the older adult worldwide (1–3). Epidemiological studies have revealed that anxiety and depression are linked to a range of adverse health outcomes, including coronary heart diseases, stroke, and even all-cause mortality (4–6). Moreover, these mental health disorders can induce neuropathic symptoms, diminish interest in daily activities, and impair memory (7, 8). However, owing to insufficient attention given to precursors of mental health issues, such as anxiety and depression, psychological disorders among the older adult population are frequently overlooked and underdiagnosed.

In recent years, research on the relationship between Body Mass Index (BMI) and mental health has attracted widespread attention (9). A systematic review shows that there exists a bidirectional association between a higher BMI and depression. Elevated BMI serves as a predictor for depression onset, while depression exacerbates the risk of overweight and obesity (10). On the other hand, obesity/overweight individuals exhibit a higher frequency of anxiety episodes compared to those of normal weight (11). Furthermore, obesity-Induced cellular senescence drives anxiety (12). Similarly, underweight individuals are more likely to experience depressive/anxiety symptoms compared to those of a normal weight. Previous studies have indicated that abnormal BMI (including obesity and underweight) is associated with an increased risk of psychological disorders such as depression and anxiety. However, the conclusions drawn from various studies remain inconsistent (13–16). A meta-analysis shows that overweight in males is associated with a reduced incidence of depression, whereas in females, this relationship exhibits a trend towards increased risk (13). Additionally, a study observed a U-shaped relationship between anxiety and BMI among whites. Blacks showed a strong association between anxiety and obesity, whereas no such association was found among Asians or Hispanics (15). In summary, this suggests that factors such as gender, race, culture, and social background may influence the relationship between BMI and depression and anxiety. Another possible explanation lies in the existence of potential mediating variables in the relationship between BMI and anxiety/depression symptoms.

A meta-analysis indicates that an increase in BMI is associated with limitations in Activities of Daily Living (ADL) (17). Numerous studies have also found a significant correlation between older adults’ ADL and Instrumental Activities of Daily Living (IADL) with symptoms of anxiety and depression (18–20). According to the stress process theory, when the BMI of older adult individuals is either excessively high or low, it can lead to a decline in their ADL and IADL abilities. The resulting stress may then contribute to cognitive impairment-related diseases, such as depression (21). Therefore, ADL and IADL can be viewed as a type of mediating variable. A study has demonstrated that ADL and IADL impairments mediate the relationship between cognitive impairments, depressive symptoms, and the physical component score (22). Additionally, ADL also mediates the relationship between BMI and life satisfaction (23). However, as of now, there’s no hard evidence indicating that ADL and IADL serve as mediators between BMI and the symptoms of anxiety and depression. Hence, we hypothesize that ADL and IADL play a certain role in the relationship between BMI and the manifestations of anxiety and depression.

Previous studies have already demonstrated the impact of abnormal BMI on anxiety and depression symptoms, and several investigations have also examined disparities within genders and age groups. Despite this, the specific dynamics of this relationship within the older adult population in China still await comprehensive exploration. Furthermore, whether ADL and IADL mediate this effect remains unexplored. Meanwhile, due to the complexity of psychological issues faced by the older adult, which are often influenced by multiple factors, it is imperative to conduct research discussing the relevant influencing factors and mechanisms to promote their mental health. Based on these compelling reasons, our study aims to investigate the impact of BMI on anxiety and depression symptoms, while also exploring the differences in different subgroups, and furthermore, investigating the potential chain mediating effects of ADL and IADL between BMI and symptoms of anxiety and depression.

## 2 Materials and methods

### 2.1 Data source and participants

**Figure 1:**
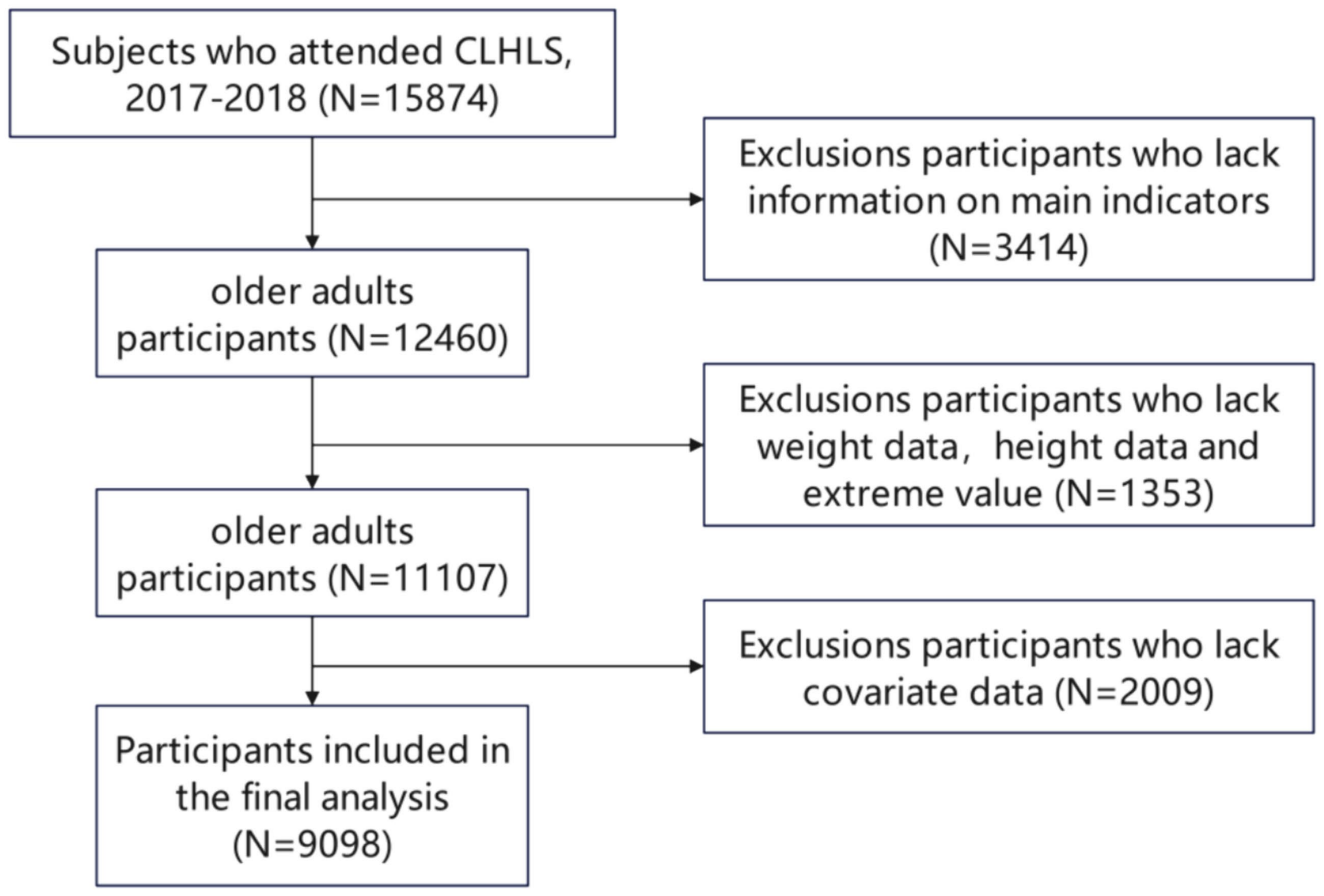
CLHLS database research participants screening flow chart.(DATA screening from top to bottom as indicated by the arrow).

The dataset utilized in this study was derived from the China Longitudinal Healthy Longevity Survey (CLHLS), a nationally representative survey conducted by the Center for Healthy Aging and Development at Peking University. The survey, which began in 1998 and has conducted 8 surveys with follow-ups every 3–4 years, used a multi-stage cluster random sampling method, covering more than half of the country’s regions (23 provinces/cities/autonomous regions). Face-to-face interviews were conducted with older adults aged 65 and above. Questions that could not be reliably answered by the oldest participants due to potential limitations such as low educational levels and/or poor hearing and vision were excluded from the survey (24). All participating respondents voluntarily signed the informed consent form during the survey. For older adult individuals unable to sign, family members signed the informed consent form on their behalf. To represent the current conditions among older individuals, we utilized the dataset from the 2017–2018 CLHLS, encompassing 15,874 participants. We excluded participants who did not complete the 10-item Center for Epidemiologic Studies Depression Scale (CES-D-10) and the 7-item Chinese Generalized Anxiety Disorder (GAD-7) scale, as well as those with missing or extreme values for height and weight data. Concurrently, after excluding individuals with incomplete data on pertinent covariates, we finalized a sample size of 9,098 older adults for our study. The specific screening steps are illustrated in Figure 1.

### 2.2 Depression

The CLHLS study adopted the CES-D-10 questionnaire to assess depressive symptoms experienced by respondents during the previous week. This questionnaire has been validated for use among older adult individuals in China, demonstrating good reliability with a Cronbach α coefficient of 0.78–0.79 (25). The CES-D-10 scale comprises 10 questions targeting specific emotions or behaviors. Points are assigned according to the reported duration of negative emotions or behaviors: 0 points if lasting less than a day, 1 point for 1 to 2 days, 2 points for 3 to 4 days, 3 points for 5 to 6 days, and 4 points for a full week. Notably, for questions 5, 7, and 10, the scoring pattern is inverted. Based on the application research of CES-D-10 in Chinese older adult individuals (26), we defined the dichotomous depression variable with a cutoff score of 12 or above.

### 2.3 Anxiety

In the CLHLS questionnaire, anxiety was assessed using the GAD-7 scale, focusing on symptoms experienced over the past 2 weeks. The GAD-7 showed excellent internal consistency (Cronbach’s α = 0.92) and good test–retest reliability (intraclass correlation = 0.83) (27). The GAD-7 scale comprises seven items, each scored on a four-point Likert scale: never (0), several days (1), more than half the days (2), and almost every day (3). The total score, ranging from 0 to 21, reflects the severity of anxiety, with higher scores indicating greater anxiety. Consistent with prior research, a GAD-7 score of 5 or above indicates the presence of anxiety.

### 2.4 Body mass index

Based on Chinese standards (28), BMI was calculated for each participant using the formula: BMI = weight (kg) / (height (m))^2. The participants were then divided into four BMI classes according to their calculated BMI values: underweight (BMI < 18.5), normal weight (18.5 ≤ BMI < 24), overweight (24 ≤ BMI < 28), and obesity (BMI ≥ 28).

### 2.5 Functional health

ADL disability was assessed using Katz index scale, which is evaluated based on six items: bathing, dressing, indoor transfer, toileting, eating, and continence. The IADL scales assessment includes the following eight items: visiting neighbors, shopping, cooking, washing clothes, walking for 2 kilometers continuously, lifting 5 kilograms, squatting three times continuously, and taking public transportation alone. Previous evidence has shown that ADL and IADL can be used as independent indicators for effectively predicting functional disability in the older adult (29, 30). If the respondent “can complete it by themselves,” it is coded as 1, “with some difficulty” is coded as 2, and “cannot” is coded as 3; the scoring range for ADL is 0–18, and for IADL it is 0–24.

### 2.6 Covariates

We controlled for the following confounding factors (16, 31–36): sociodemographic characteristics (age (65–79/ ≥ 80), gender (female/male), residence area (city/town/rural), co-residence (with family/alone/nursing room), marital status (married/others), education (no school education/≥1 year), income source (pensions/family/others) and economics status (higher/medium/lower), lifestyle (tea consumption (everyday/often/hardly), smoking (yes/no), drinking (yes/no), exercise (yes/no)), chronic disease (none/1/≥2)).

We classified education as no formal education or more than 1 year of education. Current marital status was classified as married or other (divorced/widowed/never married). Subjects were categorized as current smoker or non-current smoker and current drinker or non-current drinker. Current participation in purposive fitness activities was classified as regular exercise. The number of chronic diseases (such as Hypertension, Diabetes, Heart disease, Stroke, cerebrovascular disease, Bronchitis, asthma, pneumonia, Pulmonary tuberculosis, Cancer, and Others) was recorded through self-reporting.

### 2.7 Statistical analysis

Frequency with percentage was used to describe categorical variables. The baseline characteristics were summarized based on depressive and anxiety symptoms, and the differences between participants with and without depressive or anxiety symptoms were analyzed using the chi-square test.

To examine the association between BMI and symptoms of anxiety and depression, logistic regression models were used to calculate odds ratio (OR) with 95% confidence interval (CI). We constructed three logistic models: the unadjusted model (Model 1); Model 1 adjusted for age, gender, residence area, co-residence, marital status, education, income source, and economic status (Model 2); Model 2 with additional adjustment for tea consumption, alcohol, smoking, exercise, and the number of chronic diseases (Model 3). We performed a set of subgroup analyses to explore whether associations varied by gender, age, marital status, and residence area.

To demonstrate whether there exist a series of multiple mediation effects of BMI and symptoms of anxiety or depression between ADL and IADL, we used SPSS macro PROCESS program (Model 6) designed by (37) for data analysis. A *p*-value <0.05 was considered statistically significant. We set the bootstrap confidence interval at 95% with a bootstrap sample size of 5,000. If zero was not included in the interval of 95% CI, it indicated a significant mediation effect.

## 3 Results

### 3.1 General characteristics

Among the 9,098 participants, 1,074 cases (11.8%) exhibited anxiety symptoms, of whom 541 were classed as underweight according to their BMI. Similarly, 1,458 cases (16.0%) exhibited depressive symptoms, of whom 749 were classed as underweight according to their BMI. Factors such as BMI, age, gender, residence area, co-residence, education, income source, economic status, tea consumption, alcohol, smoking, exercise, and chronic diseases were significant determinants of anxiety and depression symptoms (*p* < 0.05). Marital status was a significant determinant of depression only (*p* < 0.05). Refer to Table 1 for further details.

**Table 1:** Demographic characteristics and anxiety/depressive symptoms in older adult (*N* (%)).

Characteristic	Anxiety		*P* value	Depression		*P* value
	Yes (*n* = 1,074)	No (*n* = 8,024)		Yes (*n* = 1,458)	No (*n* = 7,640)	
BMI			0.002			<0.001
Underweight	541 (11.5)	4,181 (88.5)		749 (15.9)	3,973 (84.1)	
Normal weight	248 (10.9)	2,035 (89.1)		331 (14.5)	1,952 (85.5)	
Overweight	88 (11.3)	689 (88.7)		106 (13.6)	671 (86.4)	
Obesity	197 (15.0)	1,119 (85.0)		272 (20.7)	1,044 (79.3)	
Age (years)			0.03			<0.001
65–79	489 (12.7)	3,374 (87.3)		533 (13.8)	3,330 (86.2)	
≥80	585 (11.2)	4,650 (88.8)		925 (17.7)	4,310 (82.3)	
Gender			<0.001			<0.001
Male	375 (8.9)	3,840 (91.1)		546 (13.0)	3,669 (87.0)	
Female	699 (14.3)	4,184 (85.7)		812 (18.7)	3,971 (81.3)	
Residence area			<0.001			<0.001
City	198 (8.0.8)	2,056 (91.2)		296 (13.1)	1958 (86.9)	
Town	396 (13.2)	2,608 (86.8)		526 (17.5)	2,478 (82.5)	
Rural	480 (12.5)	3,360 (87.5)		636 (16.6)	3,204 (83.4)	
Co-residence			0.015			<0.001
With family	829 (11.3)	6,494 (88.7)		1,082 (14.8)	6,241 (85.2)	
Alone	207 (13.8)	1,296 (86.2)		314 (20.9)	1,189 (79.1)	
Nursing home	38 (14.0)	234 (86.0)		62 (22.8)	210 (77.2)	
Marital status			0.088			<0.001
Married	582 (12.4)	4,126 (87.6)		879 (18.7)	3,829(81.3)	
Others	492 (11.2)	3,898 (88.8)		579 (13.2)	3,811 (86.8)	
Education			<0.001			<0.001
No	577 (14.6)	3,368 (85.4)		788 (20.0)	3,157 (80.0)	
Yes	497 (9.6)	4,656 (90.4)		670 (13.0)	4,483 (87.0)	
Income source income			<0.001			<0.001
Pensions	231 (8.6)	2,440 (91.4)		321 (12.0)	2,350 (88.0)	
Family	544 (13.1)	3,600 (86.9)		760 (18.3)	3,384 (81.7)	
Others	299 (13.1)	1,984 (86.9)		377 (16.5)	1,906 (83.5)	
Economic status			<0.001			<0.001
Higher	120 (6.4)	1,741 (93.6)		128 (6.9)	1,733 (93.1)	
Medium	715 (11.2)	5,654 (88.8)		1,006 (15.8)	5,363 (84.2)	
Lower	239 (27.5)	629 (72.5)		324 (37.3)	544 (62.7)	
Tea consumption			0.03			<0.001
Everyday	176 (10.1)	1,561 (89.9)		223 (12.8)	1,514 (87.2)	
Often	58 (10.7)	484 (89.3)		81 (14.9)	461 (85.1)	
Hardly	840 (12.3)	5,979 (87.7)		1,154 (16.9)	5,665 (83.1)	
Alcohol consumption			<0.001			<0.001
No	958 (12.5)	6,723 (87.5)		1,310 (17.1)	6,371 (82.9)	
Yes	116 (8.2)	1,301 (91.8)		148 (10.4)	1,269 (89.6)	
Smoking			<0.001			<0.001
No	941 (12.4)	6,677 (87.6)		1,273 (16.7)	6,345 (83.3)	
Yes	133 (9.0)	1,347 (91.0)		185 (12.5)	1,295 (87.5)	
Exercise			<0.001			<0.001
No	754 (12.9)	5,091 (87.1)		1,140 (19.5)	4,705 (80.5)	
Yes	320 (9.8)	2,933 (90.2)		318 (9.8)	2,935 (90.2)	
Chronic disease status			<0.001			<0.001
None	251 (9.7)	2,341 (90.3)		345 (13.3)	2,247 (86.7)	
1	310 (11.0)	2,518 (89.0)		398 (14.1)	2,430 (85.9)	
≥ 2	513 (13.9)	3,165 (86.1)		715 (19.4)	2,963 (80.6)	

### 3.2 Association between BMI and the symptoms of anxiety and depression symptoms

As shown in Tables 2, 3, Model 1 indicates that, in comparison to individuals with normal BMI, underweight is a significant factor of anxiety and depression symptoms (OR = 1.361, *p* < 0.001; OR = 1.382, *p* < 0.001). After controlling for demographic and socioeconomic characteristics (Model 2), underweight remains a significant influencing factor affecting anxiety and depression symptoms (OR = 1.257, *p* = 0.014; OR = 1.180, *p* = 0.045). In the fully adjusted model (Model 3), underweight remains a significant influencing factor affecting anxiety and depression symptoms (OR = 1.316, *p* = 0.004; OR = 1.232, *p* = 0.013).

**Table 2:** Multivariate logistic regression analysis of the association between BMI and anxiety symptoms.

Characteristic	Reference	Model1		Model2		Model3	
		OR [95%CI]	*P* value	OR [95%CI]	*P* value	OR [95%CI]	*P* value
BMI							
Underweight	Normal weight	1.361 [1.141–1.622]***	<0.001	1.257 [1.047–1.511]*	0.014	1.316 [1.094–1.582]**	0.004
Overweight	Normal weight	0.942 [0.803–1.105]	0.461	0.974 [0.826–1.149]	0.756	0.932 [0.789–1.100]	0.404
Obesity	Normal weight	0.987 [0.777–1.254]	0.915	0.974 [0.762–1.246]	0.837	0.905 [0.706–1.159]	0.429
Age (years)							
≥80	65–79			0.753 [0.641–0.884]***	<0.001	0.734 [0.625–0.863]***	<0.001
Gender							
Female	Male			1.560 [1.341–1.814]***	<0.001	1.414 [1.202–1.664]***	<0.001
Residence area							
Town	City			1.309 [1.050–1.631]*	0.017	1.407 [1.125–1.761]**	0.003
Rural	City			1.175[0.940–1.468]	0.156	1.253[0.999–1.571]	0.051
Co-residence							
Alone	With family			1.141 [0.951–1.370]	0.156	1.153 [0.960–1.385]	0.128
Nursing home	With family			1.479 [1.023–2.137]*	0.038	1.455 [1.005–2.107]*	0.047
Marital status							
Married	Others			1.070 [0.905–1.265]	0.428	1.059 [0.896–1.253]	0.501
Income source							
Family	Pensions			0.997 [0.805–1.235]	0.978	1.058 [0.851–1.316]	0.612
Others	Pensions			0.948 [0.755–1.190]	0.643	1.005 [0.798–1.266]	0.966
Economic status							
Medium	Higher			1.657 [1.350–2.033]***	<0.001	1.670 [1.360–2.051]***	<0.001
Lower	Higher			4.806 [3.751–6.158]***	<0.001	4.635 [3.612–5.948]***	<0.001
Education							
Yes	No			0.767 [0.655–0.899]***	<0.001	0.762 [0.649–0.893]***	<0.001
Tea consumption							
Often	Everyday					0.947 [0.686–1.306]	0.739
Hardly	Everyday					0.905 [0.752–1.089]	0.290
Alcohol							
Yes	No					0.785 [0.631–0.977]*	0.030
Smoking							
Yes	No					0.865 [0.697–1.073]	0.186
Exercise							
Yes	No					0.865 [0.746–1.004]	0.056
Chronic disease							
< 1	None					1.198 [1.000–1.434]*	0.049
≥ 2	None					1.702 [1.435–2.018]***	<0.001

*Model 1:crude model; Model 2: adjusted age, gender, residence area, co-residence, marital status, income source, economic status and education; Model 3: further adjusted tea consumption, alcohol, smoking, exercise and chronic diseases based on Model2, *p* ≤ 0.05*, *P* ≤ 0.01**, *P* ≤ 0.001***.

**Table 3:** Multivariate logistic regression analysis of the association between BMI and depression symptoms.

Characteristic	Reference	Model1		Model2		Model3	
		OR [95%CI]	*P* value	OR [95%CI]	*P* value	OR [95%CI]	*P* value
BMI							
Underweight	Normal weight	1.382 [1.184–1.613]***	<0.001	1.180 [1.003–1.388]*	0.045	1.232 [1.046–1.453]*	0.013
Overweight	Normal weight	0.899 [0.782–1.035]	0.139	1.024 [0.884–1.186]	0.754	0.994 [0.856–1.153]	0.932
Obesity	Normal weight	0.838 [0.673–1.043]	0.114	0.918 [0.732–1.152]	0.460	0.851 [0.677–1.071]	0.169
Age (years)							
≥80	65–79			1.151 [0.997–1.328]	0.056	1.065 [0.920–1.233]	0.398
Gender							
Female	Male			1.324 [1.159–1.512]***	<0.001	1.167 [1.011–1.347]*	0.035
Residence area							
Town	City			1.132 [0.934–1.372]	0.206	1.190 [0.975–1.452]	0.087
Rural	City			1.013 [0.835–1.230]	0.894	1.043 [0.854–1.274]	0.681
Co-residence							
Alone	With family			1.264 [1.081–1.479]**	0.003	1.306 [1.114–1.531]***	<0.001
Nursing home	With family			1.730 [1.272–2.354]***	<0.001	1.734 [1.268–2.370]***	<0.001
Marital status							
Married	Others			0.913 [0.787–1.059]	0.229	0.907 [0.781–1.054]	0.202
Income source							
family	Pensions			1.067 [0.883–1.289]	0.505	1.102 [0.906–1.340]	0.333
Others	Pensions			0.940 [0.767–1.152]	0.550	0.967 [0.784–1.193]	0.755
Economic status							
Medium	Higher			2.404 [1.978–2.921]***	<0.001	2.367 [1.944–2.882]***	<0.001
Lower	Higher			7.546 [5.963–9.549]***	<0.001	7.034 [5.544–8.924]***	<0.001
Education							
Yes	No			0.843 [0.733–0.970]*	0.017	0.861 [0.747–0.993]*	0.040
Tea consumption							
Often	Everyday					1.052 [0.790–1.400]	0.731
Hardly	Everyday					0.956 [0.807–1.131]	0.598
Alcohol							
Yes	No					0.712 [0.584–0.868]***	<0.001
Smoking							
Yes	No					0.907[0.750–1.096]	0.311
Exercise							
Yes	No					0.520 [0.452–0.599]***	<0.001
Chronic disease							
< 1	None					1.140 [0.970–1.339]	0.111
≥ 2	None					1.888 [1.622–2.198]***	<0.001

*Model 1:crude model; Model 2: adjusted age, gender, residence area, co-residence, marital status, income source, economic status and education; Model 3: further adjusted tea consumption, alcohol, smoking, exercise and chronic diseases based on Model2, *P* ≤ 0.05*, *P* ≤ 0.01**, *P* ≤ 0.001***.

### 3.3 Association between BMI and the symptoms of anxiety and depression symptoms in different subgroups

As shown in Tables 4, 5, in different subgroups, underweight remains a significant influencing factor affecting anxiety and depression symptoms. Among older adult individuals with underweight, males (OR = 1.565; OR = 1.554), those aged 80 and above (OR = 1.366; OR = 1.296), unmarried individuals (OR = 1.227; OR = 1.316), and those residing in town (OR = 1.526; OR = 1.427) exhibited a higher risk.

**Table 4:** Multivariate logistic regression analysis of the association between BMI and anxiety symptoms in different subgroups.

Model	Normal	Underweight		Overweight		Obesity	
		OR [95%CI]	*P* value	OR [95%CI]	*P* value	OR [95%CI]	*P* value
Gender							
Male	1 (ref)	1.565 [1.137–2.154]**	0.006	1.030 [0.790–1.341]	0.829	0.937 [0.612–1.436]	0.766
Female	1 (ref)	1.203 [0.959–1.509]	0.110	0.866 [0.698–1.074]	0.191	0.883 [0.651–1.199]	0.425
Age (years)							
65–79	1 (ref)	1.244 [0.860–1.798]	0.246	0.948 [0.756–1.189]	0.644	0.817 [0.587–1.137]	0.230
≥80	1 (ref)	1.366 [1.100–1.696]**	0.005	0.896 [0.696–1.153]	0.393	1.025 [0.702–1.497]	0.899
Marital status							
Married	1(ref)	1.344 [0.976–1.851]	0.070	0.945 [0.752–1.188]	0.628	0.922 [0.658–1.294]	0.640
Others	1(ref)	1.316 [1.048–1.653]*	0.018	0.927 [0.725–1.185]	0.543	0.890 [0.616–1.285]	0.534
Residence area							
City	1 (ref)	1.062 [0.642–1.757]	0.814	0.798 [0.559–1.138]	0.213	0.599 [0.337–1.063]	0.080
Town	1 (ref)	1.526 [1.132–2.058]**	0.006	1.026 [0.776–1.357]	0.855	0.952 [0.629–1.441]	0.816
Rural	1 (ref)	1.294 [0.988–1.696]	0.061	0.921 [0.711–1.193]	0.534	1.096 [0.756–1.589]	0.630

* Adjusted age, gender, education, income source, marital status, economic status, residence area, co-residence, tea consumption, alcohol, smoking and exercise and chronic diseases, *P* ≤ 0.05*, *P* ≤ 0.01**, *P* ≤ 0.001***.

**Table 5:** Multivariate logistic regression analysis of the association between BMI and depression symptoms in different subgroups.

Model	Normal	Underweight		Overweight		Obesity	
		OR [95%CI]	*P* value	OR [95%CI]	*P* value	OR [95%CI]	*P* value
Gender							
Male	1 (ref)	1.554 [1.188–2.032]**	0.001	0.949 [0.749–1.202]	0.665	0.776 [0.523–1.150]	0.207
Female	1 (ref)	1.081 [0.878–1.332]	0.464	1.008 [0.830–1.224]	0.936	0.876 [0.659–1.164]	0.361
Age (years)							
65–79	1 (ref)	1.058 [0.735–1.524]	0.762	0.879 [0.703–1.098]	0.257	0.887 [0.649–1.212]	0.451
≥80	1 (ref)	1.296 [1.076–1.562]**	0.006	1.104 [0.901–1.351]	0.339	0.751 [0.530–1.063]	0.106
Marital status							
Married	1 (ref)	1.292 [0.959–1.741]	0.092	0.962 [0.773–1.197]	0.726	0.943 [0.683–1.303]	0.723
Others	1 (ref)	1.227 [1.007–1.495]*	0.043	1.044 [0.850–1.283]	0.683	0.761 [0.547–1.058]	0.104
Residence area							
City	1 (ref)	0.903 [0.569–1.431]	0.663	0.954 [0.706–1.290]	0.761	0.895 [0.574–1.395]	0.624
Town	1 (ref)	1.427 [1.095–1.859]**	0.009	1.060 [0.818–1.373]	0.658	1.004 [0.680–1.484]	0.983
Rural	1 (ref)	1.205 [0.947–1.533]	0.128	0.962 [0.761–1.216]	0.746	0.729 [0.500–1.062]	0.100

* Adjusted age, gender, education, income source, marital status, economic status, residence area, co-residence, tea consumption, alcohol, smoking and exercise and chronic diseases, *P* ≤ 0.05*, *P* ≤ 0.01**, *P* ≤ 0.001***.

### 3.4 Mediation analysis

**Figure 2:**
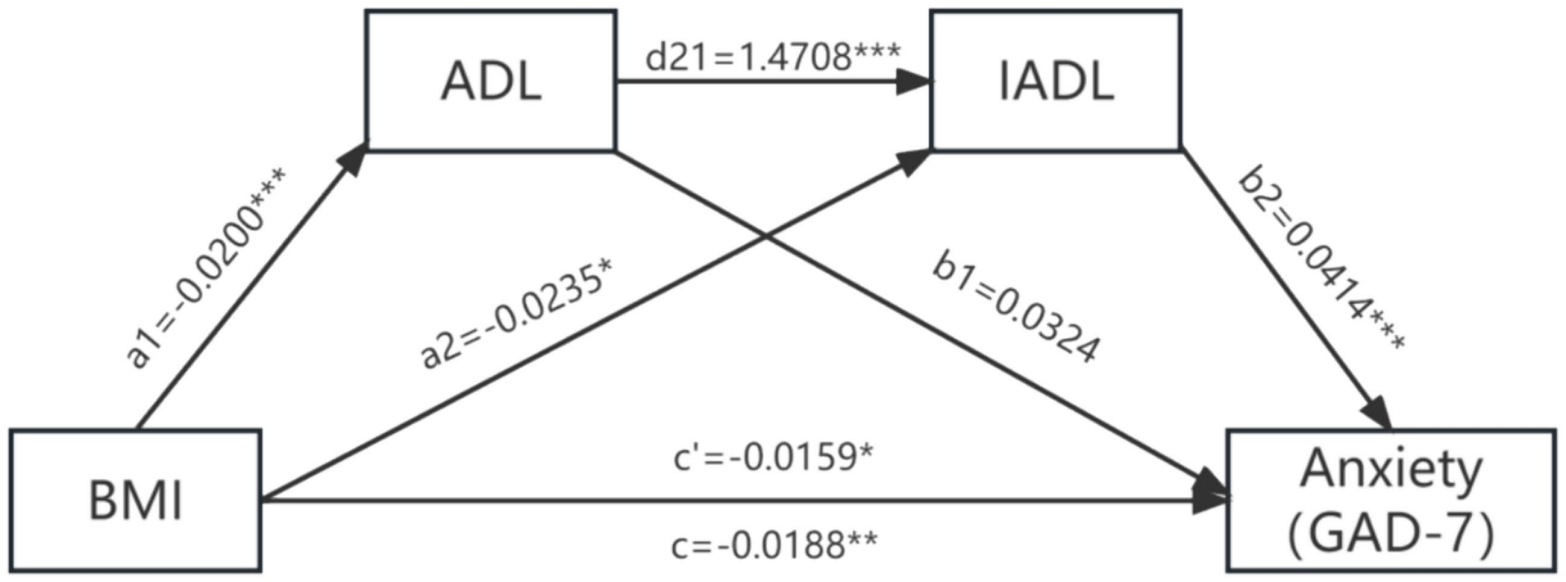
The mediation model of ADL and IADL between BMI and anxiety symptoms.

**Figure 3:**
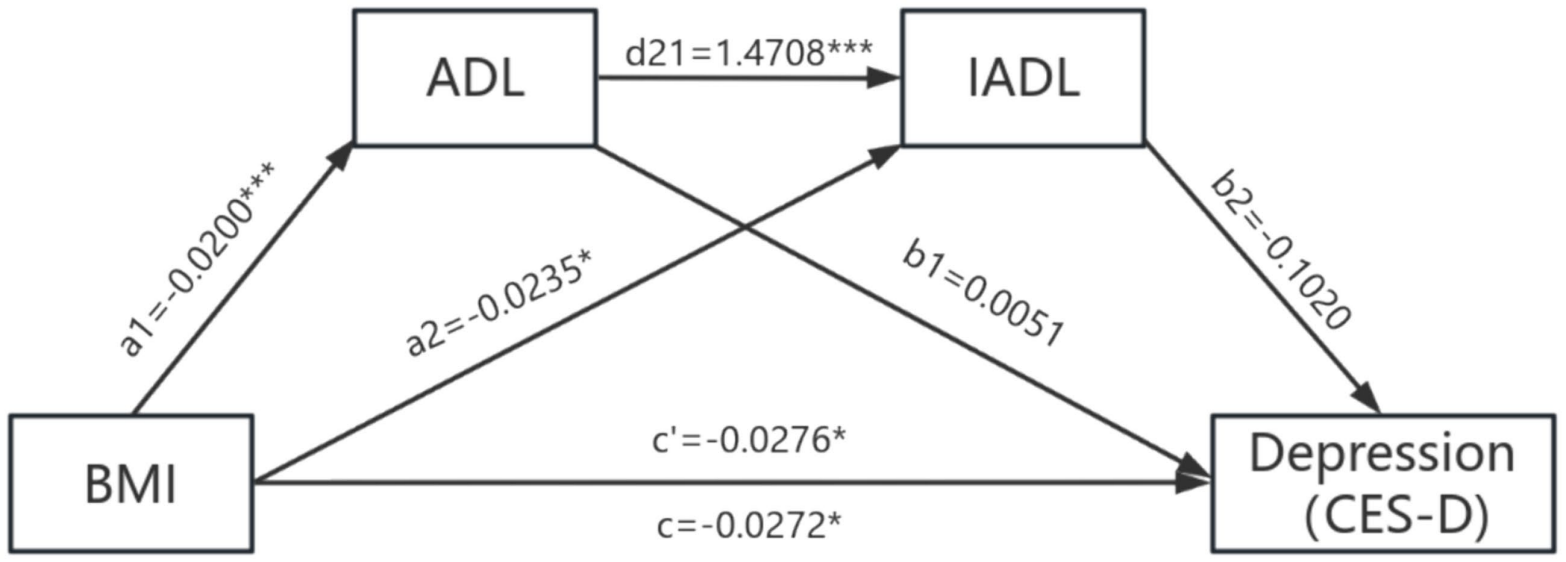
The mediation model of ADL and IADL between BMI and depression symptoms.

After controlling for covariates, ADL and IADL played a chain-mediated role between BMI and anxiety symptoms in the older adult. BMI not only had a direct effect on anxiety symptoms in the older adult (effect = −0.0159; SE = 0.0066; 95%CI: LL = −0.0288, UL = −0.0031), but also influenced them indirectly through two pathways: the independent mediating role of IADL (effect = −0.0010; SE = 0.0005; 95%CI: LL = −0.0018, UL = −0.0003) and the chain-mediated role of ADL and IADL (effect = −0.0012; SE = 0.0004; 95%CI: LL = −0.0020, UL = −0.0006). The detailed mediation analysis is shown in Table 6 and Figure 2. ADL and IADL do not mediate the relationship between BMI and depressive symptoms in older adult individuals is shown in Table 7 and Figure 3.

**Table 6:** The mediation model of ADL and IADL between BMI and anxiety symptoms.

Pathway	Effect	SE	BootLLCI	BootULCI
Total effect (c)	−0.0188	0.0066	−0.0317	−0.0059
Direct effect (c’)	−0.0159	0.0066	−0.0288	−0.0031
a1	−0.0200	0.0041	−0.0280	−0.0119
a2	−0.0235	0.0092	−0.0416	−0.0055
d21	1.4708	0.0236	1.4246	1.5170
b1	0.0324	0.0201	−0.0070	0.0718
b2	0.0414	0.0075	0.0268	0.0561
Indirect effects				
Total indirect effects	−0.0028	0.0008	−0.0045	−0.0014
Indirect 1	−0.0006	0.0005	−0.0018	0.0003
Indirect 2	−0.0010	0.0005	−0.0020	−0.0002
Indirect 3	−0.0012	0.0004	−0.0020	−0.0006

Indirect 1, BMI→ADL→anxiety; Indirect 2, BMI→IADL→anxiety; Indirect 3, BMI→ADL→IADL→anxiety. BootLLCI, bootstrapping lower limit confidence interval; BootULCI, bootstrapping upper limit confidence interval; SE, standard error; Effect, standardized regression coefficient.

**Table 7:** The mediation model of ADL and IADL between BMI and depression symptoms.

Pathway	Effect	SE	BootLLCI	BootULCI
Total effect (c)	−0.0272	0.0110	−0.0488	−0.0056
Direct effect (c’)	−0.0276	0.0110	−0.0493	−0.0060
a1	−0.0200	0.0041	−0.0280	−0.0119
a2	−0.0235	0.0092	−0.0416	−0.0055
d21	1.4708	0.0236	1.4246	1.5170
b1	0.0051	0.0338	−0.0611	0.0712
b2	−0.0102	0.0126	−0.0349	0.0144
Indirect effects				
Total indirect effects	0.0004	0.0007	−0.0008	0.0018
Indirect 1	−0.0001	0.0007	−0.0014	0.0013
Indirect 2	0.0002	0.0003	−0.0004	0.0010
Indirect 3	0.0003	0.0004	−0.0004	0.0011

Indirect 1, BMI→ADL→depression; Indirect 2, BMI→IADL→depression; Indirect 3, BMI→ADL→IADL→depression. BootLLCI, bootstrapping lower limit confidence interval; BootULCI, bootstrapping upper limit confidence interval; SE, standard error; Effect, standardized regression coefficient.

## 4 Discussion

The main purpose of this study was to explore the relationship between BMI and anxiety or depression symptoms among the older adult in China, as well as investigate whether ADL (activities of daily living) and IADL (instrumental activities of daily living) can moderate the relationship between BMI and anxiety/depression symptoms. First, our research results showed a significant association between underweight and anxiety/depression symptoms. Second, among older adult individuals with underweight, males, those aged 80 and above, unmarried individuals, and those residing in urban areas exhibited a higher risk. Third, ADL and IADL played a chained mediating role in the relationship between BMI and anxiety symptoms among the older adult.

This study found that among the older adult population in China, underweight is an independent predictor of symptoms of depression and anxiety. Unlike some overseas studies (13, 36), the effect of overweight and obesity on depression and anxiety was not statistically significant compared with normal BMI. In other words, BMI levels did not show a U-shaped relationship with anxiety and depression among the older adult population in China. There is an old Chinese saying that “a broad mind brings a plump body,” which means that a person with a good mood will look calm and thus gain weight. A British survey study has proved this point, showing that the possibility of depression and anxiety is lower for the older adult as BMI increases (38). On the other hand, people with better diet quality that is rich in vegetables, lean meat, fish, and whole grains are less likely to suffer from emotional disorders or anxiety (39, 40). In addition, another possible explanation is response shift (41), which means that obese people may have adapted to their condition and begun to positively evaluate their mental health.

The study results indicate that among older adult individuals with underweight, males, those aged 80 and above, unmarried individuals, and those residing in urban areas exhibit a higher risk compared to those with a normal BMI. Older adult males are more likely to experience symptoms of anxiety and depression when they lose weight, a phenomenon that has also been reported in other studies (42–44). This may be related to the traditional Chinese concept that obesity represents a good social and economic status (45), On the other hand, it could also be associated with dieting, as unhealthy eating patterns are more likely to cause anxiety and depression (46, 47). In our study, there was no significant correlation between underweight or obesity among female participants. This is contrary to the findings of studies in Japan and Korea (48, 49), which may be related to social status (50). Married women tend to shoulder more family responsibilities (51), and work–family conflicts or interference are more likely to lead to psychological distress (52), ultimately resulting in anxiety and depression.

In terms of age, older adult individuals over the age of 80 are more prone to psychological issues, which may be related to their self-care abilities and emotional well-being. Research has shown that older adults require more financial support, daily care, and emotional support as they age (53).

In terms of marital status, consistent with previous studies (54–56), single and unmarried older adult individuals are more likely to experience symptoms of anxiety and depression. Single older adult individuals generally lack social interactions, and spouses are the primary source of support for the older adult (47). Receiving care from spouses can reduce the risk of depression among the older adult (57).

In our study, living in urban areas is more likely to lead to anxiety and depression symptoms, possibly because of the relatively stable economic level and abundant social resources in cities (58). Meanwhile, the living environment in rural areas can effectively reduce stress (59). Conversely, people living in urban areas are more likely to be affected by issues related to social resources, resulting in anxiety (35).

The study revealed that ADL and IADL play a chain-mediated role between BMI and anxiety symptoms among the older adult. Specifically, BMI is first negatively correlated with ADL, which then leads to increased limitations in IADL, ultimately exacerbating the risk of anxiety. Prior research has shown that weight loss is associated with a decrease in bone mineral content and bone density, which increases the risk of osteoporosis and fractures, resulting in ADL limitations among the older adult (60). On the other hand, underweight also leads to muscle loss and reduced muscle strength, which are significant factors contributing to ADL limitations among the older adult (61). There is also a negative correlation between underweight and IADL, as a higher BMI represents a better living standard, potentially leading to better access to medical resources, which may be beneficial for IADL function (62). Firstly, limitations in IADL increase dependence on others, thereby leading to increased anxiety in daily life (63). On the other hand, ADL and IADL impairments restrict social activities among the older adult. Previous studies have shown that more social activities can help older adult individuals achieve better mental health (64). Participating in social activities not only provides positive interpersonal interaction opportunities for the older adult, but also enriches their social life and greatly enhances their confidence in obtaining support when needed. This interpersonal interaction can effectively prevent and alleviate interpersonal issues, while an enhanced sense of support makes the older adult more resilient in the face of life adversities, reducing sensitivity to negative experiences (65), ultimately lowering their risk of anxiety (66).

Moreover, ADL/IADL impairments also have a significant impact on the participation of older adult individuals in physical activities. Regular participation in physical activities can lead to positive emotional experiences (64), thereby reducing mental health problems, which is also one of the strategies for treating anxiety and depression (67, 68).

This study possesses numerous strengths, among them a considerable and nationally representative sample of community-dwelling older Chinese adults, distinguished by a significant inclusion of the oldest-old demographic. Moreover, the CLHLS, encompassing 23 provinces or municipalities across China, covers regions that display a wide range of geographical, economic, public resource, and health characteristics, thereby offering a robust and comprehensive representation of the Chinese population. The questionnaire design that is based on international standards while also being adapted to the cultural and social context of China. Furthermore, our study adds to the limited literature on the relationship between BMI and anxiety/depression symptoms among older adult individuals in China.

However, this study also has some limitations. Firstly, our research design was a cross-sectional study, which cannot determine the causal relationship between variables. Secondly, it would be better to explore the impact of BMI on anxiety and depression among older adult individuals across different subgroups in future studies. Thirdly, other potential mediating or moderating factors could also be further studied. Finally, although some studies have shown that the data we used has good reliability, these results mainly rely on the self-evaluation of the respondents.

## 5 Conclusion

In conclusion, despite some limitations in our study, it still provides guidance on current health issues among the older adult. The findings indicate a significant correlation between underweight and anxiety/depression symptoms among Chinese older adult individuals. This suggests that relevant departments can take measures to reduce emaciation among the older adult, such as nutritional support, daily care, financial assistance and psychological support. These measures can further prevent ADL and IADL limitations among the older adult and ultimately reduce their risk of developing anxiety and depression symptoms.

## Data Availability

Publicly available datasets were analyzed in this study. This data can be found here: https://opendata.pku.edu.cn/dataverse/CHADS.
